# Brown adipogenic potential of brown adipocytes and peri-renal adipocytes from human embryo

**DOI:** 10.1038/srep39193

**Published:** 2016-12-16

**Authors:** Nan-Nan Wu, Chuan-Hai Zhang, Hyuek-Jong Lee, Yan Ma, Xin Wang, Xiao-Juan Ma, Wei Ma, Dong Zhao, Ying-Mei Feng

**Affiliations:** 1 Beijing Key Laboratory of Diabetes Prevention and Research, Lu He hospital, Capital Medical University, Beijing 101149, China; 2 Department of Endocrinology, Lu He hospital, Capital Medical University, Beijing 101149, China; 3 Key Laboratory of Animal Ecology and Conservation Biology, Institute of Zoology, Chinese Academy of Sciences, Beijing 100101, China; 4 Beijing Advanced Innovation Center for Food Nutrition and Human Health, College of Food Science and Nutritional Engineering, China Agricultural University, Beijing, 100083, China; 5 Department of Gynaecology and Obstetrics, Lu He hospital, Capital Medical University, Beijing 101149, China

## Abstract

Both brown adipocytes (BAC) and beige cells hold therapeutic potential for the treatment of metabolic disorders. Unfortunately, the amount and activity of these cells are limited in adults. Although BAC marker expression has been shown in peri-renal adipose tissues in children and adults, functional assessment is lacking. Furthermore, it is entirely unknown whether adipose progenitors are present in human embryo and able to give rise to BAC *in situ* during evolution. Therefore, adipose tissues in the interscapular and peri-renal regions were dissected from human embryo and subcutaneous white adipose tissues (sWAT) were obtained from an adult. After subjected to differentiation *in vitro,* adipocyte progenitors were detected present in all these adipose tissues. When stimulated for adipogenesis, differentiated adipocytes in the intercapular and peri-renal regions showed similar features: (1) induced BAC and beige cell marker expression including UCP1 and PRDM16 and comparable mitochondrion copy number; (2) similar gene expression patterns by RNA-Seq analysis; and (3) similar maximal oxygen consumption rates examined by respirometry. Nevertheless, stimulation of adipocyte progenitors in sWAT induces neither BAC and beige cell marker expression nor any change of oxygen consumption. In conclusion, peri-renal adipocyte progenitors in human embryo hold browning potential for BAC production.

The prevalence of obesity is increasing rapidly in the past 20 years worldwide^1^. As the major risk factor for cardiovascular diseases and metabolic disorders, obesity develops when the energy intake exceeds the energy consumption^2^,^3^. In adult mammals, white and brown adipose tissues are mainly composed by brown or white adipocytes, respectively. Accumulated evidence have shown that brown adipocytes (BAC) and white adipocytes (WAC) are derived from different precursors and featured as distinct morphology^4^,^5^,^6^. BAC contain multilocular lipid droplets and a much higher number of mitochondria than WAC^7^. The mitochondria of BAC uncouple large amounts of fuel oxidation from ATP for the generation of heat^8^. In contrast, WAC are involved in energy storage.

Conventionally, brown adipose tissues (BAT) are believed to be present exclusively in small mammals and human infants^9^. Quantification of BAT activity in human by positron-emission tomography (PET)–computed tomography (CT) using radiotracers such as ^18^F-fluorodeoxyglucose (^18^F-FDG) reveals that active BAT tissues are present in adults and inversely associated with body-mass index^10^,^11^,^12^. In line with these findings, BAT transplantation enhances whole-body energy metabolism, reduces obesity and improves glucose tolerance and insulin sensitivity in a variety of murine models^13^,^14^,^15^. These data consistently indicate that increase of BAT amount and/or activity could be an ideal approach for the treatment of obesity and metabolic abnormalities. Unfortunately, both the amount and the activity of BAT are dramatically decreased in adults^16^.

Recently, another subset of adipocytes, so called “beige cells”, was identified. They are located within white adipose tissues (WAT) in adults and could be activated by cold temperature and norepinephrine for mitochondrion genesis^17^,^18^. Activated BAC and beige cells not only share common signatures in gene expression profiles^17^,^19^ but also carry similar protective properties against obesity by improving whole-body energy metabolism, increasing triglyceride clearance and attenuating of insulin resistance^14^,^20^. To be noted, although BAC and beige cells share similar functions, they are indeed derived from different progenitors^21^,^22^. Consequently, the regulatory machinery of BAC and beige cells might differ upon activation. For instance, global deletion of PRDM16 leads to abnormal BAT morphology. However, specific deletion of PRDM16 in postnatal adipose tissues using adiponectin-Cre impedes browning of subcutaneous WAT but has minimal effects on BAT^23^.

Recent studies reported the expression of BAT markers in peri-renal WAT in children^24^, healthy adults^25^,^26^, and patients with pheochromocytoma^27^ or hypertension^28^. Nevertheless, functional assessment was done only in one group but failed to detect any difference in oxygen consumption using differentiated mesenchymal stem cells isolated from peri-renal adipose tissues^25^. Therefore, whether BAC exist in peri-renal WAT is not well defined. Furthermore, it is yet unknown whether progenitor cells are present in neck and peri-renal regions of human embryo and give rise to BAC *in situ* during evolution. To answer the questions, we isolated cells from fat tissues located in the neck and peri-renal regions in human embryo and investigated their brown adipogenic differentiation potential, thermogenic capacity, gene signature and metabolic function *in vitro*.

## Results

### Adipocyte progenitors in different tissues of human embryo

To investigate the browning potential of adipocytes, classical interscapular BAT (cBAT) and peri-renal WAT (prWAT) were isolated from biopsy of human embryo. In parallel, subcutaneous WAT (sWAT) in the peritoneal omentum region were isolated from an adult and served as the reference control. After digestion and isolation, cells in stromal vascular fraction (SVF) of the preparations were obtained and cellular components were assessed by FACS.

**Figure 1:**
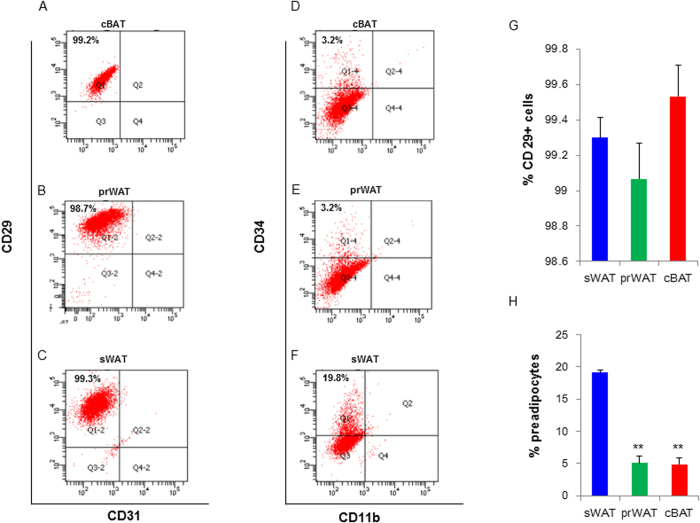
Adipocyte progenitors in different locations of human embryo. Cells in stromal vascular fraction were isolated from interscapular (cBAT) and peri-renal (prWAT) fat tissues in human embryo and subcutaneous WAT (sWAT) in an adult, respectively. They were stained with antibody cocktails against CD29, CD31, CD11b and CD34. Adipocyte progenitors, defined as CD29+CD31−CD11b−CD34+ cells were quantified by FACS. Representative FACS dot plots of CD29/CD31 of cBAT, prWAT and cWAT were shown in (**A–C**), respectively. When gated on CD29+CD31− cells, FACS dot plots of CD11b/CD34 in cBAT, prWAT and sWAT were illustrated in (**D–F**), respectively. (**G**) The percentage of CD29+ cells in cBAT, prWAT and sWAT. (**H**) The percentage of adipocyte progenitors, i.e. CD29+CD31−CD11b−CD34+ cells in cBAT, prWAT and sWAT. N = 3 per group. Significance of the difference: *p < 0.05, **p < 0.01 and ^#^p < 0.001 when compared to sWAT, respectively.

As adipogenic progenitors are defined as CD29^+^/CD31^−^/CD11b^−^/CD34^+^ expressing cells^29^,^30^,^31^, SVF cells were stained with the antibodies against these markers for FACS. As shown in Fig. 1, the percentage of CD29^+^CD31^−^ cells was comparable among cBAT, prWAT and sWAT (Fig. 1A–C). When gated on CD29^+^CD31^−^ cells, the percentage of CD11b^−^CD34^+^ cells was similar between cBAT and prWAT (Fig. 1D–F). The percentage of CD29+ cells was comparable among sWAT, prWAT and cBAT (Fig. 1G). The proportion of preadipocytes in sWAT, prWAT and cBAT, defined as CD29+ CD31− CD34+ CD11b− cells, were quantified by FACS (Fig. 1H).

Immediately after isolation, SVF cells were subjected for *in vitro* differentiation. Notably, these cells were able to expand for 8–11 passages with sustained morphology and capacity for adipogenic differentiation (data not shown). Thus, cells from passages 3 to 5 were used in the study.

### The adipogenic characteristics of cBAC and prWAC

**Figure 2:**
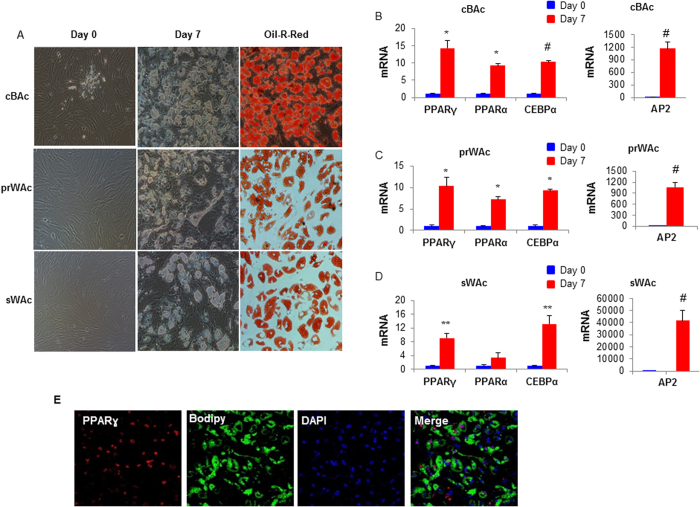
Characterization of adipogenic potential of cBAC and prWAC. In the study, adipocytes differentiated from classical BAT and peri-renal WATs in human embryo were named as cBAC and prWAC, respectively, whereas cells derived from sWAT were called sWAC. After 7 days of stimulation, cBAC, prWAC and sWAC were stained with Oil-Red-O (x200) (**A**). The expression of key adipocyte markers PPARɣ, PPARα, CEBPα and AP2 in differentiated cells were studied by real-time PCR (**B–D**). Data were expressed as fold change. cBAC, prWAC and sWAC were stained with anti-human PPARɣ and Bodipy to study PPARɣ expression. (**E**) prWAC were stained with anti-human PPARɣ and Bodipy to study PPARɣ expression (x200). N = 3 per group. Significance of the difference: *p < 0.05, **p < 0.01 and ^#^p < 0.001 when compared to sWAC, respectively.

In the study, adipocytes differentiated from cBAT and prWAT in human embryo were named as cBAC and prWAC, respectively, whereas cells derived from sWAT were called sWAC. Upon induced for brown adipogenesis by the differentiation cocktail, cells were able to differentiate into mature adipocytes as evidenced by phase contrast image and Oil-Red-O staining (Fig. 2A). Consistently, key adipogenic genes including PPARɣ, PPARα, C/EBPα and AP2 expressions were dramatically increased in cBAC, prWAC and sWAC (Fig. 2B–D). The expression of these key adipogenic genes in prWAC was further confirmed by immunostaining. The representative image of PPARɣ expression in prWAC was shown in Fig. 2E.

Collectively, these results demonstrated that adipocyte progenitor cells were present in the interscapular and peri-renal regions in human embryo with intact adipogenic potential.

### The browning potential of prWAC

**Figure 3:**
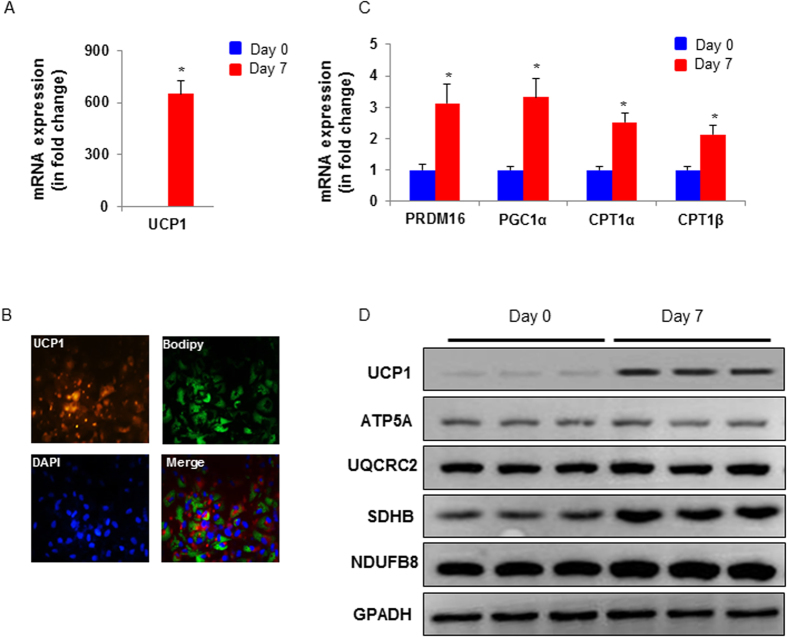
Brown potential of prWAC. After induced differentiation for 7 days, prWAC showed dramatic induction of UCP1 expression in both mRNA (**A**) and protein (**B**) levels. In parallel, a panel of brown adipocyte markers were evaluated. (**C**) mRNA expression of PRDM16, PGC1α. CPT1α and CPT1β was upregulated at day 7 compared with day 0. (**D**) Western blot data of the expression of brown adipocyte markers. N = 3 per group. Significance of the difference: *p < 0.05, **p < 0.01 and ^#^p < 0.001 when compared to sWAC, respectively.

Upon stimulation with differentiation cocktail, UCP1, the golden marker of brown adipocytes, was dramatically increased in prWAC, as quantified by its mRNA expression by real-time PCR and protein expression by immunostaining, respectively (Fig. 3A and B). Except UCP1, mRNA expression of thermogenic genes such as PRDM16, PGC1α, CPT1α and CPT1β were significantly increased (Fig. 3C). Western blot data further confirmed the upregulation of UCP1 as well as OXPHOS proteins including ATP5A, ubiquinol-cytochrome C reductase core protein II (UQCRC2), NADH (dehydrogenase (ubiquinone) 1β subcomplex) (NDUFB8) and succinate dehydrogenase complex and subunit B (SDHB) in prWAC after 7 days of stimulation (Fig. 3D).

### Comparison of the brown adipogenic potential among cBAC, prWAC and sWAC

**Figure 4:**
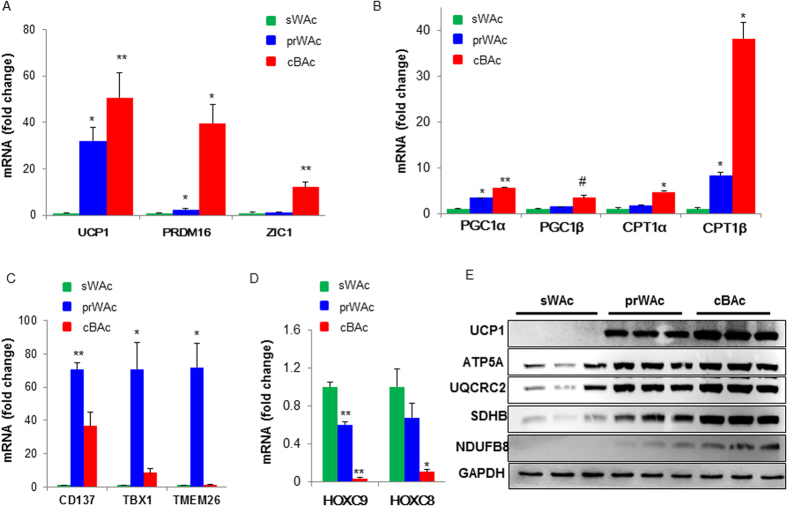
Comparison of brown potential of cBAC, prWAC and sWAC. cBAC, prWAC and sWAC were induced differentiation for 7 days by stimulation cocktail. At the end of differentiation, cells were collected and the phenotype of differentiated cells were characterized by BAC markers (**A**), fat acid oxidative related genes (**B**), beige cell markers (**C**) and WAT markers (**D**) by real-time PCR. Data were expressed as fold change. In the meantime, proteins were extracted and the expression of BAC and beige cell markers after stimulation were compared by western blot (**E**). N = 3 per group. Significance of the difference: *p < 0.05, **p < 0.01 and ^#^p < 0.001 when compared to sWAC, respectively.

Thereafter, we compared the brown adipogenic potential of cBAC, prWAC and sWAC. To this end, BAC marker genes were first analyzed by quantitative real-time PCR. Not surprisingly, the expression of UCP1, PRDM16 and Z1C1 was much higher in cBAC than sWAC (cBAC *vs*. sWAC: p = 0.003 for UCP1; p = 0.042 for PRDM16; and p = 0.001 for Z1C1) (Fig. 4A). Compared to sWAC, these genes were seen induced in prWAC although the increased expression level of Z1C1 did not reach statistical significance (prWAC *vs* sWAC: p = 0.017 for UCP1; p = 0.018 for PRDM16; and p = 0.33 for Z1C1) (Fig. 4A). Likewise, the expression of fatty acid oxidation related genes, PGC1α and PGC1β were upregulated in cBAC and prWAC when compared with sWAC (cBAC *vs*. sWAC: p = 0.009 for PGC1α and p = 0.001 for PGC1β; prWAC *vs*. sWAC: p = 0.024 for PGC1α and p = 0.058 for PGC1β) (Fig. 4B). The induction of CPT1α and CPT1β expression was 4.7- and 38.1-fold higher in cBAC than sWAC, separately (p < 0.05 for both). In parallel, CPT1α and CPT1β were 1.7- and 8.4-fold greater in prWAC than sWAC, respectively (p = 0.305 for CPT1α and p = 0.028 for CPT1β) (Fig. 4B).

Next, we analyzed the expression of CD137, TBX1 and TMEM-26 in sWAC, cBAC and prWAC. When compared to sWAC, CD137 expression was 70.9- and 36.6-fold increase in cBAC and prWAC, respectively (prWAC *vs.* sWAC: p = 0.003; cBAC *vs*. sWAC: p = 0.052). Similar as CD137, TBX1 expression was 69.9- and 8.5-fold higher in prWAC and cBAC when compared to sWAC (prWAC *vs*. sWAC: p = 0.034; cBAC *vs*. sWAC: p = 0.086). TMEM-26 expression was 71.8-fold higher in prWAC when compared to cBAC and sWAC (prWAC *vs.* sWAC: p = 0.039) (Fig. 4C).

In contrast to the induction of beige cell markers expression, WAC marker HoxC8 and HoxC9 were dramatically reduced in cBAC and prWAC (HoxC8: p = 0.024 for cBAC *vs.* sWAC and p = 0.155 for prWAC *vs*. sWAC; HoxC9: p = 0.008 for cBAC *vs*. sWAC and p = 0.031 for prWAC *vs*. sWAC) (Fig. 4D).

Thereafter, BAT and beige cell markers expression was further validated by western blot. The UCP1 and mitochondrial-specific oxphos proteins, including ATP5A, UQCRC2, SDHB and NDUFB8 were significantly increased in cBAC and prWAC (Fig. 4E). However, almost no UCP1 expression and weak oxphos expression were seen in sWAC (Fig. 4E).

### Metabolic characterization of the differentiated adipocytes

**Figure 5:**
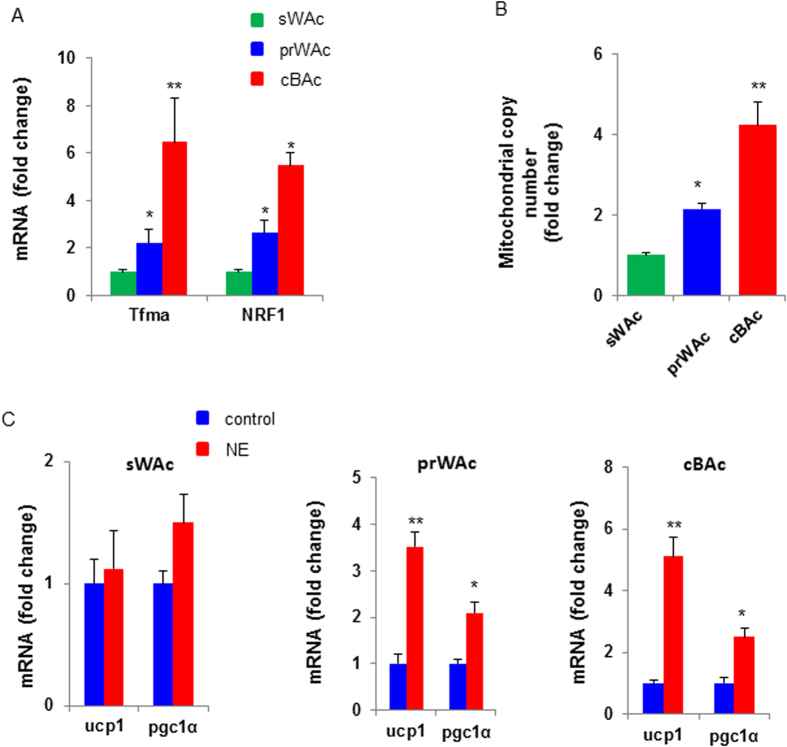
Mitochondriongenesis of prWAC. To assess mitochondrial copy number, mRNA expression of Tfam and NRF1 (**A**) and mitochondrial copy number (**B**) in cBAC, prWAC and sWAC were studied. (**C**) The effect of norepinephrine on UCP1 and PGC1α expression in cBAC, prWAC and sWAC. NE denotes norepinephrine. N = 3 per group. Significance of the difference: *p < 0.05, **p < 0.01 and ^#^p < 0.001 when compared to sWAC, respectively.

The key feature of BAC is the enriched amount of mitochondria and enhanced metabolic activity, both of which are greater than that of WAC^7^. To evaluate the function of the differentiated adipocytes, we quantified mitochondrial biogenesis related genes such as Tfam and NRF1 expression and mitochondrial copy number. Concordant to the results described above, we found that Tfam and NRF1 expression and mitochondrial copy number were significantly increased in cBAC and prWAC, both of which were higher than sWAC (Fig. 5A and B).

It is known that the sympathetic nervous system can activate BAC by releasing norepinephrine. The function of norepinephrine on BAC activation is mediated by β-adrenergic receptor (β-AR) system. Addition of norepinephrine promoted the expression of UCP1 and PGC1α in cBAC and prWAC but had limited effect on sWAC (Fig. 5C). Collectively, these data indicated that both cBAC and prWAC held brown adipogenic potential.

### RNA-Seq analysis of cBAC, prWAC and sWAC

**Figure 6:**
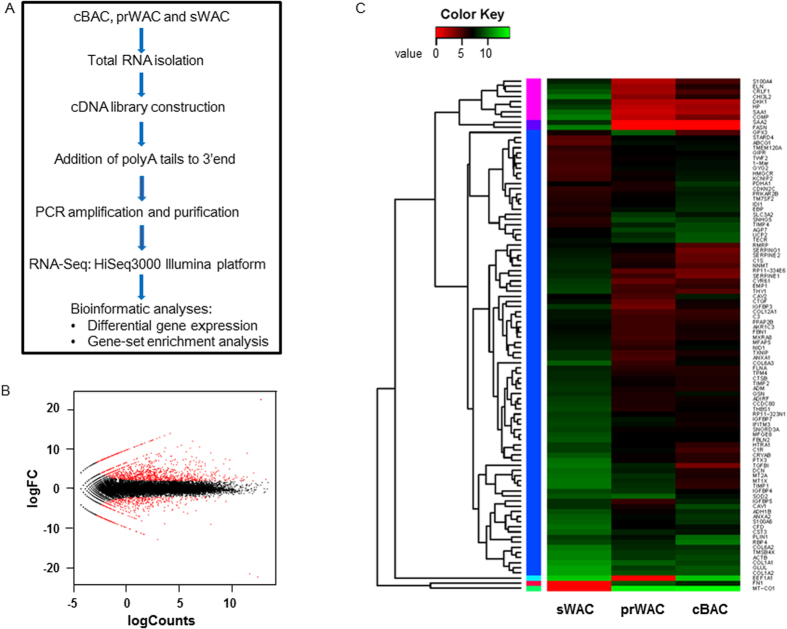
Transcriptome analysis of gene expression profile in differentiated cells. To compare gene expression profile in cBAC, prWAC and sWAC, RNA-Seq analysis was performed using RNA isolated from differentiated cells. (**A**) Graphical presentation of the methods involved. (**B**) MA plot of genes sequenced in prWAC and sWAC. The differentially expressed genes between prWAC and sWAC are in red dots. LogFC means log_2_ (fold change). Red dots on the upper part denote upregulated genes whereas red dots in the lower part indicate downregulated genes. (**C**) Heat map of gene expression patterns of cBAC, prWAC and sWAC. N = 3 per group.

To get more insight of molecular signature, total RNA were isolated from cBAC, prWAC and sWAC to perform RNA-Seq analysis. The method involved in RNA-Seq was illustrated in Fig. 6A. The differentially expressed genes were identified using the thresholds of false discovery rate (FDR) ≤ 0.05. When compared to sWAC, totaled 2005 genes were differentially expressed in cBAC among which 1548 genes were upregulated and 457 genes were downregulated. Similarly, 1722 genes were differentially expressed in prWAC when compared to sWAC, among which 1210 genes were detected downregulated and 511 genes were downregulated. Red dots in MA plot presented the differentially expressed genes between prWAC and sWAC (Fig. 6B). Heat map analysis showed transcriptome profiles of cBAC, prWAC and sWAC (Fig. 6C), indicating that prWAC and BAC shared common gene expression pattern.

**Figure 7:**
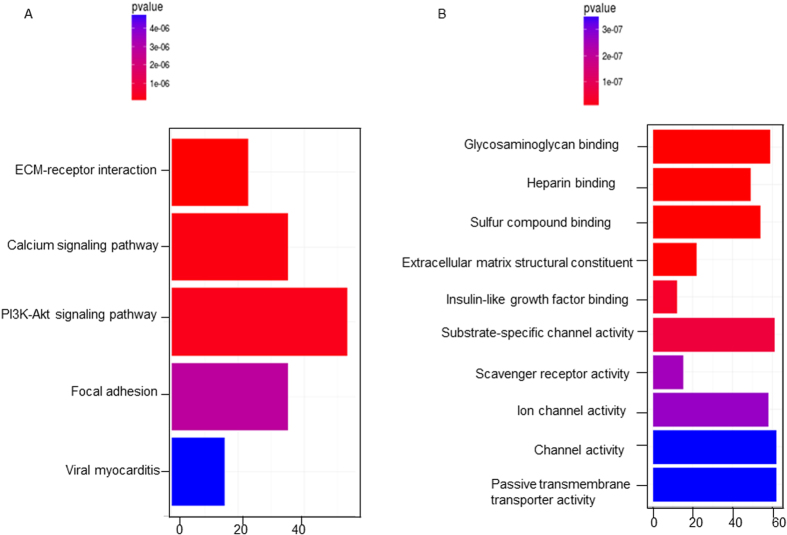
Comparison of prWAC and sWAC by RNA-Seq analysis. Pathways (**A**) and molecule functions (**B**) enriched in prWAC as compared with sWAC, which were identified by KEGG pathway analysis. N = 3.

As our main interest, we further determined which gene pathways were differentially expressed between prWAC and sWAC by GO term and KEGG pathway analysis. Pathways and molecule functions that were enriched in prWAC as compared with sWAC were identified by KEGG analysis (Fig. 7A and B).

### Functionality of the differentiated adipocytes

**Figure 8:**
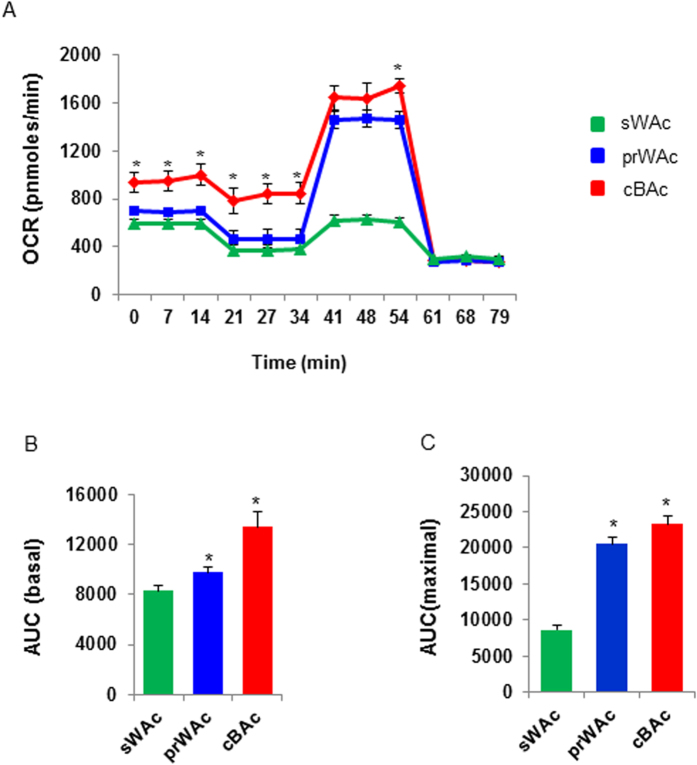
Real-time determination of mitochondrion respiration. cBAC, prWAC and sWAC were induced for differentiation for 6 days. After harvest, cells were seeded at a density of 4 × 10^5^ cells/cm^2^ in XF24 V7 cell culture microplates and allowed for differentiation for another 24 hours. Cell medium was taken to quantify oxygen consumption rate by A Seahorse Bioscience XF24 Extracellular Flux Analyzer. After measuring baseline level of oxygen consumption rate (OCR), cells were stimulated with 1 μmol/L FCCP to assess the maximum OCR in the cell culture. Areas under the curve (AUC) was calculated with the software provided by the manufacturer. (**A**) Baseline and maximum levels of OCR were expressed as a function of time. (**B**) Baseline level of OCR was presented as AUC. (**C**) Maximum level of OCR was presented as AUC. The experiments were performed in triplicates and oxygen concentration in the cell medium was quantified every seven minutes. N = 6–7 per group. Significance of the difference: *p < 0.05, **p < 0.01 and ^#^p < 0.001 when compared to sWAC, respectively.”

It is well known that mitochondrion number is largely different between BAC and WAC. As oxygen consumption rate (OCR) serves as an indicator of mitochondrial respiration, we examined OCR in cBAC, prWAC and sWAC using A Seahorse Bioscience XF24 Extracellular Flux Analyzer. cBAC, prWAC and sWAC were induced for differentiation for 6 days as described above. After harvest, cells were seeded at a density of 4 × 10^5^ cells/cm^2^ and continued differentiation for another 24 hours. Oxygen concentration in cell medium was measured before and after stimulation. The basal level of OCR was higher in cBAC and prWAC when compared with sWAC. After stimulation, OCR was accelerated to increase in cBAC and prWAC but not in sWAC (Fig. 8A). The maximum OCR was comparable between cBAC and prWAC, however, respiratory capacity of sWAC remained poor in the entire experiments. When OCR was calculated and presented as the area under the curve (AUC), the basal level of respiration rates of prWAC and cBAC were 1.2- and 1.6-fold higher than sWAC, respectively (p < 0.05 for both). Similarly, the maximal respiration rates of prWAC and cBAC were 2.4- and 2.7-fold greater than that of sWAC (p < 0.05 for both) (Fig. 8B and C).

Put together, prWAC were potent to give rise to brown adipocyte-like cells and could be an alternative candidate for BAC production.

## Discussion

In the study, we demonstrated that (1) bonafide brown adipocyte progenitors could be obtained from human embryo with high differentiation ability; (2) adipocyte progenitor cells from embryonic peri-renal fat tissues had brown adipogenic potential as evidenced by the enriched mitochondrial number, BAC marker expression, common transcriptome profiles and metabolic function when compared to classical BAC.

As a new avenue of obesity treatment, how to generate large amount of functional BAC has attracted great interest for cell therapy. The key obstacle is that BAT in human adults have heterogeneous activity^6^. Second, the source of BAT is rare and adult humans do not have bonafide BAT like mouse. To overcome these limitations, several important attempts have been made on the search for other source of BAC. For example, genetic overexpression of PPARγ, C/EBPα and PRDM16 genes mediated trans-differentiation of induced pluripotent stem cells (iPS) cells toward BAC^32^ and inhibition of Janus kinase activity facilitated conversion of WAC into BAC in iPS-derived adipocytes^33^. As the consequence of these efforts, isolation, cultivation and characterization of human BAC have been achieved^6^,^29^. In spite of promising progress in BAT field, the safety and efficacy of iPS-derived BAC are still under evaluation and cell therapy is still far from clinical application.

It is well known that BAT are distributed in infants and young children widely but disappeared or lost activity in adults. Looking for new sources of BAT and beige cells is of interest. Recently, studies reported the browning adipose marker expression detected in peri-renal adipose tissues in children and adults but there is no evidence proving whether these cells are functional. Thus, whether adipocytes in prWAT could serve as BAC is yet unclear.

Previously, we and others demonstrated that BAT transplantation could ameliorates obesity^13^,^14^,^15^. In the current study, we showed that prWAC had high and similar properties as classical BAC in the aspects of gene expression profiles, induced BAT activity by norepinephrine, increased number of mitochondria and enhanced metabolic activity. In addition, we verified here that BAC derived from human embryo displayed bonafide brown adipocyte characteristics.

After induction by activation cocktail, we observed different expression fold of TBX1 and TMEM26 in cBAC and prWAC when compared to sWAC. Likewise, the expression of HoxC8 and HoxC9 was reduced in different amplitude in cBAC and prWAC. Although the activation of classical BAT and the browning process could be induced by common mechanisms (eg, noradrenergic-mediated induction by cold), brown adipocytes present in white adipose tissue (WAT) derive from precursors different from those in classical brown adipose tissue (cBAT) and are closer to the white adipocyte cell lineage^34^. For instance, BAT morphology was adversely affected in PRDM16-null mice but not in mice with specific deletion of PRDM16 in postnatal adipose^23^. Moreover, Scheele *et al*. reported that human-derived supraclavicular BAT represented a type of BAT with distinct features from both subcutaneous white/brite and interscapular brown fat^35^. These data collectively suggest that brown adipocytes could be heterogeneous by different locations and the regulatory machineries of brown and beige adipocytes might be intricate and different. Our data supported the notions in that the induced gene expression in prWAC and cBAC might slightly differ as they were derived from different origin. More detailed molecular analysis of cBAC and prWAC is needed for future clinical application.

In summary, our data add new values to the current knowledge in that (1) BAC do exist in peri-renal adiposes as evidenced by the expression of browning adipose tissue and beige cells markers and enhanced oxygen consumption after activation and (2) BAC appear in peri-renal region from embryonic stage till adult stage in one’s life span. They have potent differentiation ability to generate functional brown adipocytes with sufficient amount and therefore, could be a new strategy to treat obesity and its related metabolism diseases.

## Materials and Methods

### Isolation of fat tissues from human embryo

Human embryonic brown fat tissues and peri-renal fat tissues were obtained from spontaneousabortion (11 weeks of gestation) in the Department of Gynaecology and Obstetrics in Lu He hospital. Adipose tissues were dissected from neck and peri-renal of the embryo. In parallel, subcutaneous fat tissues were obtained from a colon cancer patient in the Department of Surgery in LuHe Hospital. Fetal parents and patients were free of cardiovascular disease, endocrine diseases, metabolic disorders and acute infection. Isolation of primary adipocytes from adipose tissues of human embryo and adult subcutaneous fat was performed as described previously^36^. Briefly, immediately after dissection, freshly resected fat depots were collected, minced and digested using collagenase 1 (2 mg/ml in PBS with the addition of 3.5% BSA; Worthington Biochemical Corporation, Lakewood, NJ) and the stromal vascular fraction (SVF) was isolated. Floating adipocytes were separated from the SVF by centrifugation at 300 × g for 3 min.

The study complied with the Helsinki Declaration for investigation of human subjects. It received ethical approval from the competent Institutional Review Boards of the Capital Medical University. All participants provided written informed consent.

### Cultivation of primary human embryo adipocytes

After isolation, SVF cells were plated and grown in high-glucose Dulbecco’s modified Eagle’s medium (DMEM/H) supplemented with 20% (vol/vol) fetal bovine serum (FBS) (Sigma-Aldrich) and 1% penicillin-streptomycin. For adipocyte differentiation, cells were grown to reach 100% confluence and then exposed to adipogenic induction mixture in DMEM/H medium containing 0.5 mM isobutylmethylxanthine, 0.1 μM dexamethasone, 0.5 μM human insulin (Sigma-Aldrich, Dallas, TX), 2 nM T3, 30 μM indomethacin, 17 μM pantothenate, 33 μM biotin and 2% FBS for 6–7 days. Adipocyte differentiation medium was refreshed every 2 days in the entire experiments.

To induce BAC activity, at the end of differentiation, cells were stimulated with 1 μM norepinephrine for 4 hours. Cells were collected for RNA extraction or western blot.

### Flow Cytometry

Cells in SVF fraction were stained with the antibody cocktails for 10 min on ice: APC-conjugated anti-human CD29 (San Diego, CA, clone TS2/16), FITC-conjugated anti-human CD11b (MiltenyiBiotec, 130–081–201), PE-conjugated anti-human CD31 (MiltenyiBiotec, 130-091-610) and PerCP-Cy5.5-conjugated anti-human CD34 (BDPharmingen). Adipocyte progenitors defined as CD29+/CD31−/CD11b−/CD34+ cells were quantified by FACS (BD FACS Aria, BD Biosciences, CA, USA). Data analysis was performed using BD FACS Diva software (BD Biosciences, CA, USA).

### Real-time PCR

Total RNA was isolated using the RNeasy Mini Kit (Qiagen). cDNA was synthesized using random hexamers (Invitrogen, Carlsbad, CA,USA) for subsequent real-time quantitative PCR analysis (ABI Prism VIIA7; Applied Biosystems Inc, Foster City, CA,USA) according to the manual. PCR products were detected using Sybr Green and normalized by cyclophilin expression. Primers were designed using Primer Quest (Integrated DNA Technologies, Inc, Coralville, IA, USA). Primer sets for quantitative real-time PCR were summarized in Table 1.

### Western blotting

Cell and tissue lysates were extracted using RIPA buffer (150 mM sodium chloride, 1.0% TritonX-100, 0.5% sodium deoxycholate, 0.1% SDS, 50 mMTris, protease and phosphatase inhibitor cocktail (Roche Diagnostics Corp, Pleasanton, CA, USA). Protein concentrations were measured by a BCA assay kit (Pierce Diagnostics Corp, Pleasanton, CA, USA). Equal amount of protein was separated by 10% SDS-PAGE, transferred to PVDF membrane (Millipore Billerica, MA, USA). After blocked in 5% skim milk in TBST (0.02 M Tris base, 0.14 M NaCl, 0.1% Tween 20, pH 7.4), membranes were incubated with primary antibodies for overnight at 4 °C and then probed with secondary antibodies conjugated with HRP (DAKO). Primary antibodies used in this study were anti-human UCP1 (Abcam, co, Cambridge, MA, USA), anti-human PPARγ, anti-human PGC-1α, anti-human OXPHOS (Abcam, co, Cambridge, MA, USA) and anti-human GAPDH (Cell Signaling Technology, Beverly, MA, USA). Signals were detected with Super Signal West Pico Chemiluminescent Substrate (Pierce, Rockford, IL, USA).

### Oil-Red-O staining

To detect neutral lipid, cells were stained with 0.2% (w/v) Oil-Red O (Sigma–Aldrich, St. Louis, MO, USA) for 10 min at room temperature after fixation with 4% paraformaldehyde. Stained cells were studied under light microscopy (LSM 780, ZEISS, Germany).

### Immunocytochemistry

To assess the expression of brown adipogenic markers, differentiated cells were stained with anti-human UCP1 at the concentration of 1 μg/ml and then stained with Alexa 488-conjugated secondary antibody (Invitrogen), BODIPY (Thermo) and DAPI (Leagene) following the instruction. Cells positive for both UCP1 and BODIPY were determined to be brown adipocytes. Cells stained with secondary antibody with the omission of primary antibody were served as negative controls. Images were taken by Zeiss laser scanning confocal microscopy (LSM780, Germany).

### Measurements of oxygen consumption

Cells were allowed for differentiation for 6 days. After collection, they were seeded (4 × 10^5^ cells/cm^2^) in XF24 V7 cell culture microplates (Seahorse Bioscience) and cultured in DMEM with 20% FBS and antibiotics (100 units/mL of penicillin and 100 μg/mL of streptomycin) overnight at 37 °C under an atmosphere of 5% CO_2_. The next day, cells were continued to undergo differentiation for 24 hours as described above. Equal amount of cell culture medium (22 μl) was taken to measure O_2_ consumption by a Seahorse Bioscience XF24 extracellular flux analyzer (XF24, Seahorse Bioscience). Basal respiration was assessed in control cells. The maximum respiratory capacity was assessed by 1 μmol/L FCCP stimulation. Finally, mitochondrial respiration was blocked with 1 μmol/L rotenone and the residual oxygen consumption rate (OCR) was obtained as non-mitochondrial respiration. Areas under the curve (AUC) was calculated with the software provided by the manufacturer (XF24, Seahorse Bioscience).

### RNA-Sequencing

To compare the gene expression among cBAC, prWAC and sWAC, total RNA was isolated from two biological replicates using RNeasy Mini Kit (QIAGEN). Equal amount (1 μg) of RNA samples were used to construct a cDNA library following the manual instruction (The NEBNext Ultra Directional RNA Library Prep Kit for Illumina (NEB, USA). Quality control was performed by Agilent 2200 TapeStation (Agilent Technologies, USA). The libraries were subjected for sequencing on an Illumina HiSeq3000 sequencer (Illumina, USA). Raw reads generated by Illumina HiSeq3000 were subjected to quality control. Sequence reads were pre-processed by the pipeline of RiboBio CO., Ltd (Beijing, China) to remove adaptors and filter low quality reads. The differential gene expression analysis was performed using the R package edgeR^37^ and differentially expressed genes were identified using the following thresholds: false discovery rate (FDR) ≤ 0.05. Gene-set enrichment analysis (GSEA) was performed to detect significantly enriched gene sets (also called pathways) using the R package clusterProfiler (version 3.0.5)^38^.

In more details, the gene name (symbol) was mapped to ncib: ENTREZID and then annotated to KEGG ID according to the mapping relationship between ENTREZID and KEGG ID. Based on the relationship between KEGG ID and KEGG pathway, the gene was mapped to the corresponding pathway KEGG pathway. In order to determine whether there was any differentially expressed gene present in the KEGG pathway identified by enrichment analysis, hypergeometric distribution model was applied which was based on the number of differentially expressed genes in different pathways and the quantity of background genes. By doing so, enrichment pathways were obtained. (q < =0.05).

Hypergeometric distribution model:


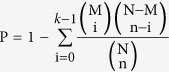


In this equation, *N* is the total number of genes in the background distribution, *M* is the number of genes within that distribution that are annotated (either directly or indirectly) to the node of interest, *n* is the size of the list of genes of interest and *k* is the number of genes within that list which are annotated to the node. The background distribution by default is all the genes that have annotation. P-values were adjusted for multiple comparison and q-values were also calculated for FDR control.

### Statistical analysis

Data were expressed as mean ± SD. Statistics analysis was performed using GraphPad Prism (GraphPad Software Inc, La Jolla, CA, USA). One-way ANOVA with Dunnett was used when comparing prWAC, cBAC against sWAC. To further study the difference between prWAC, cBAC versus sWAC, unpaired, 2-tailed Student’s t test was used for data with normal distribution. For data that did not fit normal distribution, nonparametric Mann Whitney analysis was used. A *P* value less than 0.05 was considered significant.

## Additional Information

**How to cite this article**: Wu, N.-N. *et al*. Brown adipogenic potential of brown adipocytes and peri-renal adipocytes from human embryo. *Sci. Rep.* **6**, 39193; doi: 10.1038/srep39193 (2016).

**Publisher's note:** Springer Nature remains neutral with regard to jurisdictional claims in published maps and institutional affiliations.

## Figures and Tables

**Table 1:** Lists of primers used in the study.

	5′ primer sequence	3′ primer sequence
Cyclophilin	TGTGTCAGGGTGGTGACTTC	GTCTTGGCAGTGCAGATGAA
C/EBPβ	CAAGCTGAGCGACGAGTACA	AGCTGCTCCACCTTCTTCTG
UCP1	GCAGGGAAAGAAACAGCACC	CCCGTGTAGCGAGGTTTGAT
PGC1β	CAGGCAGTAGATCCTCTTCAAG	TCCTCGTAGCTGTCATACCTG
PGC1α	GCCCAGATACACTGACTACG	CTCGAGGGTTAAGGCTGTTATC
PPARɣ	GCTATCATTACGGAGTCCACG	TCGCACTTGTCATACACCAG
PPARα	ATACATAAAGTCCTTCCCGCTG	GGGTGATGTGTTTGAACTTGATT
PRDM16	TTCGGATGGGAGCAAATACTG	CACGGATGTACTTGAGCCAG
CPT1β	ATCCTACTCCTATGACCCCG	TCTGCATTGAGACCCAACTG
mt-tRNA	CACCCAAGAACAGGGTTTGT	TGGCCATGGGTATGTTGTTA
CPT1A	CCTCCAGTTGGCTTATCGTG	TTCTTCGTCTGGCTGGACAT
AP2	CATGTGCAGAAATGGGATGG	AACTTCAGTCCAGGTCAACG
CEBP/α	ACTAGGAGATTCCGGTGCCT	GAATTCTCCCCTCCTCGCAG
TFAM	CCATATTTAAAGCTCAGAACCCAG	CTCCGCCCTATAAGCATCTTG
NRF1	GAGGCGCTGGAATGAACAAG	AGGAACACAGCAAACACCCT
CD137	AGCTGTTACAACATAGTAGCCAC	TCCTGCAATGATCTTGTCCTCT
TBX1	ACGACAACGGCCACATTATTC	CCTCGGCATATTTCTCGCTATCT
TMEM26	ATGGAGGGACTGGTCTTCCTT	CTTCACCTCGGTCACTCGC
ZIC1	AAGATCCACAAAAGGACGCA	CACGTGCATGTGCTTCTTG
Hoxc9	CGGCAGCAAGCACAAAGAG	CGGCAGCAAGCACAAAGAG
Hoxc8	ACCGGCCTATTACGACTGC	TGCTGGTAGCCTGAGTTGGA
